# Dhb Microcystins Discovered in USA Using an Online Concentration LC–MS/MS Platform

**DOI:** 10.3390/toxins11110653

**Published:** 2019-11-10

**Authors:** Johnna A. Birbeck, Nicholas J. Peraino, Grace M. O’Neill, Julia Coady, Judy A. Westrick

**Affiliations:** Department of Chemistry, Wayne State University, Detroit, MI 48202, USA; jbirbeck@chem.wayne.edu (J.A.B.); nperaino@chem.wayne.edu (N.J.P.); goneill04@gmail.com (G.M.O.); jccoady@oakland.edu (J.C.)

**Keywords:** microcystins, cyanotoxins, (2*S*,3*S*,4*E*,6*E*,8*S*,9*S*)-3-amino-9-methoxy-2,6,8-trimethyl-10-phenyldeca-4,6-dienoic acid (Adda)-ELISA, LC–MS/MS, HRMS

## Abstract

Based on current structural and statistical calculations, thousands of microcystins (MCs) can exist; yet, to date, only 246 MCs have been identified and only 12 commercial MC standards are available. Standard mass spectrometry workflows for known and unknown MCs need to be developed and validated for basic and applied harmful algal bloom research to advance. Our investigation focuses on samples taken in the spring of 2018 from an impoundment fed by Oser and Bischoff Reservoirs, Indiana, United States of America (USA). The dominant cyanobacterium found during sampling was *Planktothrix agardhii*. The goal of our study was to identify and quantify the MCs in the impoundment sample using chemical derivatization and mass spectrometry. Modifying these techniques to use online concentration liquid chromatography tandem mass spectrometry (LC–MS/MS), two untargeted MCs have been identified, [d-Asp^3^, Dhb^7^]-MC-LR and tentative [Dhb^7^]-MC-YR. [Dhb^7^]-MC-YR is not yet reported in the literature to date, and this was the first reported incidence of Dhb MCs in the United States. Furthermore, it was discovered that the commercially available [d-Asp^3^]-MC-RR standard was [d-Asp^3^, Dhb^7^]-MC-RR. This study highlights a workflow utilizing online concentration LC–MS/MS, high-resolution MS (HRMS), and chemical derivatization to identify isobaric MCs.

## 1. Introduction

The World Health Organization (WHO) provides microcystin (MC) drinking and recreational health advisory guidelines. In the last five years, the United States Environmental Protection Agency (US EPA) posted more conservative health advisory guidelines referring to the total class of MCs in drinking and recreational waters. The US EPA set a two-tier drinking water health advisory at 0.3 µg MCs/L for susceptible populations (such as young children) and 1.6 μg MCs/L for the remaining population. The recreational water health advisory is 8 µg MCs/L. Since MCs are hepatotoxins, recent studies suggest that people with non-alcoholic fatty liver disease (NAFLD) would be a susceptible population [1]. With increased incidence of NAFLD due to the rise in obesity and diabetes, the susceptible population to MC toxicity expands across all age groups. In order to determine the concentrations of all MC congeners or prevalent MCs, a robust, sensitive, untargeted analytical method needs to be developed.

**Figure 1:**
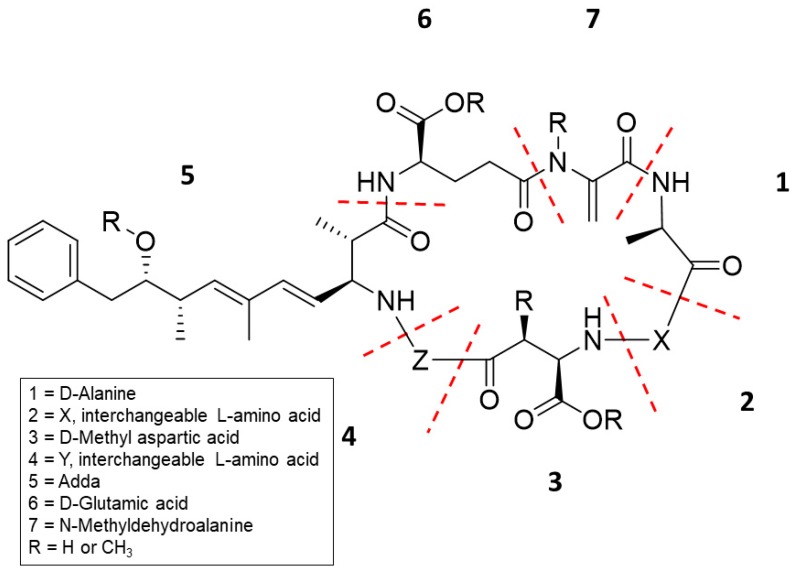
Structure of microcystin (MC) and identification of its parts. The R groups are known sites where a hydrogen or a methyl can be substituted.

MCs are cyclic heptapeptides (Figure 1) produced by over 10 genera of cyanobacteria. The most reported genera in harmful algal bloom (HAB) literature are *Microcystis*, *Dolichospermum*, *Planktothrix*, and *Nostoc*. In the advent of more sensitive and higher-resolution mass spectrometric techniques, the number of new MCs increased rapidly and is now over 246 MC congeners [2,3,4]. Figure 1 shows that positions 2 (X) and 4 (Z) can vary and are reflected in the MC congener name. MC-LA has leucine and alanine in positions 2 (X) and 4 (Z), respectively. MC has four conserved amino acids: d-methyl aspartic acid (d-MAsp); a unique cyanobacteria amino acid, (2*S*,3*S*,4*E*,6*E*,8*S*,9*S*)-3-amino-9-methoxy-2,6,8-trimethyl-10-phenyldeca-4,6-dienoic acid (Adda); d-glutamic acid (d-Glu); and methydehydroalanine (Mdha) (Figure 1). MC congener methylation/demethylation sites occur on these four conserved amino acids, and these substitutions are designated with R as shown in Figure 1. Furthermore, a seventh position substitution was reported with the presence of amino acids serine and methionine. The diversity of MC congeners gives rise to a broad range of chemical and physical properties, suggesting congener-dependent toxicokinetics and toxicodynamics.

The two most common methodologies to quantitate MCs are an Adda hapten-based enzyme-linked immunosorbent assay (Adda-ELISA) and liquid chromatography tandem mass spectrometry (LC–MS/MS) with triple quadrupole being most commonly used. The Adda-ELISA is an indirect competitive ELISA which is based on the recognition of the Adda group in position 5, and it is now a staple platform to determine the “total MC” concentration by field scientists, ecologists, and environmental scientists because the assay can be performed onsite with minimal overhead. The Adda-ELISA has cross-reactivity with the MC reference standards at 40–150% [5,6], with MC biodegradation products [7], and with MC disinfection byproducts [8,9,10]. While these cross-reactivities makes the Adda-ELISA an excellent MC screening tool, its low specificity interferes with quantitation of intact MC among byproducts, making it unsuitable as an analytical tool. LC–MS/MS can only identify and quantify MCs the method has reference standards for. The 12 commercial MC reference standards are MC-LR, MC-RR, MC-YR, MC-LA, MC-LF, MC-LW, MC-WR, MC-HtyR, MC-HilR, MC-LY, [d-Asp^3^]-MC-LR, and [d-Asp^3^]-MC-RR (Table 3). The weakness in using LC–MS/MS to determine total MC concentration is four-fold: less than 3% of known MCs have commercially available reference standards, new MCs are being discovered more frequently, most commercial reference standards are not certified, and the available standards are not verified as the most prevalent MCs in specific regions or worldwide. Therefore, both methods can commonly over- or underestimate the diversity and concentration of MCs present.

Several methodologies using high-resolution MS (HRMS) and chemical derivatization were developed to identify “untargeted” MCs. HRMS of the precursor and product ions provides chemical formulas derived with a known certainty. LC–HRMS, with accurate mass and automated data analyses, was used to determine unknown and new MCs [11,12]. Others targeted specific conserved MC MS/MS fragments [13,14,15,16,17], specifically, fragment 135 *m/z*, [Ph-CH_2_-CH(OCH_3_)], and the MH^+^ minus the neutral 135 *m/z* fragment that were observed by fast atom bombardment MS using collision-induced dissociation (FABMS/CID) [13] and electrospray ionization/CID (ESI/CID) [18] mass spectrometry. Another approach was chemical derivatization of the position 7 amino acid, Mdha/Dha [14,19]. Mdha and Dha have an α,β unsaturated carbonyl that readily undergoes a Michael’s addition in the presences of a good nucleophile such as thiol. In recent literature, several publications used chemical derivatization to discover new MCs [3,20], differentiate isomeric MCs [20], and identify MCs in complex matrices [14]. By using new technologies and understanding the strengths and weakness of each methodology, new platforms and workflows can be created to simplify untargeted MC methods.

Freshwater HABs have an increasingly negative impact on human and environmental health. Primary exposure for humans is through ingestion of drinking and recreational waters. To date, little resources were allotted for untargeted MC platform development and certified standards of the most prevalent MCs. Since toxicokinetics and toxicodynamics are MC congener-dependent, more studies are needed to determine which MCs are most prevalent and their geographic distribution. In order to execute these studies, robust, sensitive untargeted platforms are needed. The goal of this study was to determine the identity of the untargeted MCs in an impoundment sample from Indiana, United States of America (USA) using a new platform and workflow for untargeted MC analysis.

## 2. Results

### 2.1. LC–MS/MS and Adda-ELISA Quantitation

A sample from an impoundment fed by Oser and Bischoff Reservoirs in Indiana was analyzed for total MC concentration using online concentration LC–MS/MS and an Adda-ELISA kit. Online concentration LC–MS/MS is a technique which uses a dual-column system. The first column, the “concentrator” column, concentrates a large volume of the sample. The “concentrator” column is backflushed onto a second column, the “analytical” column, for separation and analysis on an MS system. Previous studies showed a discrepancy in the total MC detected using these two methods [5]. LC–MS/MS results determined three major MC variants, [d-Asp^3^]-MC-RR, MC-YR, and [d-Asp^3^]-MC-LR, which were quantified at 556 ng/L, 2731 ng/L, and 1006 ng/L, respectively. One minor MC variant MC-LY was quantified at 6 ng/L, and the total MC concentration in the impoundment sample was 4299 ng/L. The sample concentration by Adda-ELISA was 4942 ng/L MC-LR which was similar to the total MC concentration by LC–MS/MS.

In this study, the LC–MS/MS method used two criteria, chromatographic identification and quantifier to qualifier ion ratios, to verify the identification and quantification of MCs. Chromatographic identification for retention time criteria followed the US EPA method 544, LC–MS/MS method analyses of MCs and nodularin [21]. The peak retention time criterion was calculated by ± 3 standard deviations of the retention time and was calculated to be ± 0.05 min. For the qualifier/quantification transition ratio, a ± 25% criterion was used. Table 1 has the qualifier/quantification transition ratios and retention times for the MCs from the standard and the sample. The retention times observed for both [d-Asp^3^]-MC-RR and MC-YR in the impoundment sample were outside the peak retention time criteria, at −0.06 min and +0.06 min, respectively. This was not the case for [d-Asp^3^]-MC-LR with a difference of only 0.01 min (Table 1). Furthermore, the MC-YR and [d-Asp^3^]-MC-LR in the sample were not within the qualifier/quantification transition ratio range, at 51–85% and 47–79%, respectively. The reported variation in retention times between the calibrants and sample for [d-Asp^3^]-MC-RR and MC-YR was greater than the peak and the qualifier/quantification transition ratio for MC-YR and [d-Asp^3^]-MC-LR, as well as outside the ± 25% range. Using a more stringent retention time and qualifier/quantifier ratio, it was concluded that the three major MCs in the sample were not identified and quantified correctly.

### 2.2. Using HRMS to Verify MC Isomers

The impoundment sample was fractionated using high-performance liquid chromatography with a photodiode array detector (HPLC-PDA) into fractions containing the three major MCs and concentrated by N_2_ evaporation. Accurate mass by HRMS was used to determine the chemical formula of the three prevalent MCs in the sample and corresponding standards (Table 2). These results suggested that MCs in the sample had the same chemical formula as the standards of [d-Asp^3^]-MC-RR, [d-Asp^3^]-MC-LR, and MC-YR within <2 ppm. As the samples contained the same chemical formula as the standards, but with slight shifts in retention time and changing qualifier/quantification transition ratio, this suggested that the samples MCs were isomers of the standards.

### 2.3. Chemical Derivatization and HRMS Fragmentation to Identify Untargeted MCs

**Figure 2:**
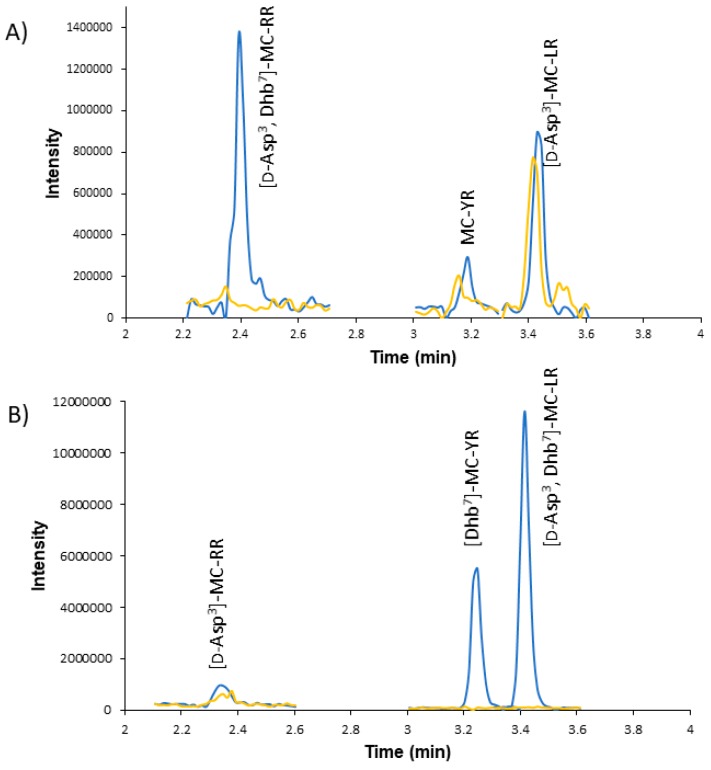
The LC–MS/MS triple quadrupole (Q3) scan shows both the unaltered MC (blue line) and the chemically derived MCs with thiol group (yellow lines). (**A**) Chromatograph of the mixture of reacted and unreacted standard MCs. MCs MC-YR and [d-Asp^3^]-MC-LR reacted with 2-mercaptoethanol, but [d-Asp^3^]-MC-RR ([d-Asp^3^, Dhb^7^]-MC-RR) did not react. (**B**) Chromatograph of the mixture of reacted and unreacted sample MCs. [d-Asp^3^]-MC-RR reacted with 2-mercaptoethanol, but MC-YR ([Dhb^7^]-MC-YR and [d-Asp^3^]-MC-LR ([d-Asp^3^, Dhb^7^]-MC-LR) did not.

Michael’s addition of 2-mercaptoethanol is a commonly used method to determine the identity of Mdha or Dha at position 7 [14,19]. In short, thiol addition indicates Mdha or Dha, while no reaction indicates either Dhb or no α,β unsaturated carbonyl. Dha and Mdhb variants would result in parent masses of one fewer or more methyl group, respectively. The impoundment sample was divided into control and experiment samples and recombined to perform online concentration LC–MS/MS in full scan mode. MC masses, as well as the addition of the 2-mercaptoethanol mass (+78), were targeted for identification. Three targeted MCs and three untargeted MCs were identified (Figure 2). In the standard sample, both [d-Asp^3^]-MC-LR and MC-YR were derivatized to the 2-mercaptoethanol conjugate, but the standard for [d-Asp^3^]-MC-RR did not. In contrast, the samples [d-Asp^3^]-MC-LR and MC-YR were not derivatized while the [d-Asp^3^]-MC-RR was. These data suggest that the standard contained [d-Asp^3^]-MC-LR, MC-YR, and [d-Asp^3^, Dhb^7^]-MC-RR while the sample contained [d-Asp^3^, Dhb^7^]-MC-LR, [Dhb^7^]-MC-YR, and [d-Asp^3^]-MC-RR. However, the possibility that [Dhb7]-MC-YR may be the constitutional isomer [D-Asp^3^-Dhb^7^]-MC-HtyR cannot be excluded.

**Figure 3:**
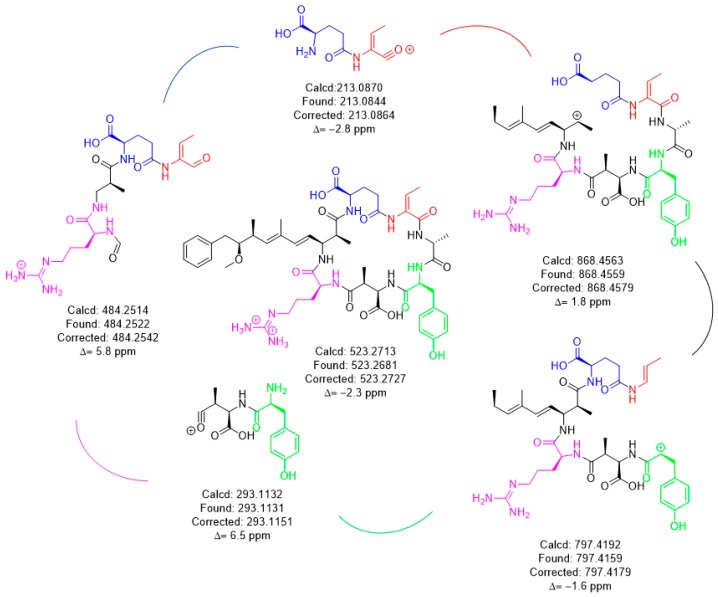
HRMS fragmentation and identification of [Dhb^7^]-MC-YR.

**Figure 4:**
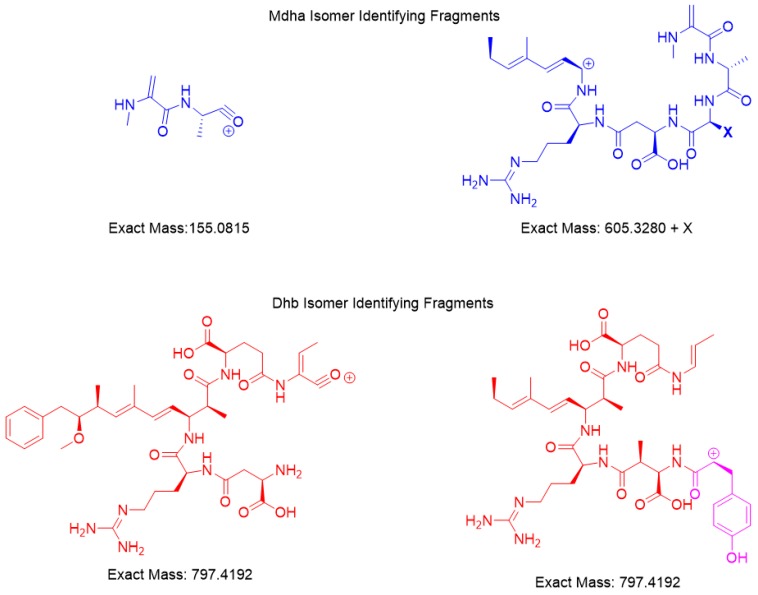
HRMS fragments unique for the [Mdha^7^] and [Dhb^7^] MCs.

HRMS with product ion scan was used to determine the structure of the isomers. Fragments identified had <7 ppm agreement with the *m/z* ion molecular formula used to derive the fragment chemical structure (Figure 3). The common fragments were produced from the [M + 2H]^+2^ *m/z* parent using a constant higher energy collisional-induced dissociation (HCD) of 15. Unique fragments of [Mdha^7^] MCs were [H + Mdha-Ala]^+^ (155.0815 *m/z*) and [Mdha-Ala-X-Masp-Arg-C_11_H_14_O]^+^ (605.3280 + X *m/z*) (Figure 4). The proposed [Dhb^7^] isomers were found to contain the unique fragments [Asp-Arg-Adda-Glu-Dhb] (797.4192 *m/z*). However, in MC-YR, the expected methylated aspartic acid fragment was not observed at 811.4349 *m/z*. In this case, 797.4192 *m/z* was observed as isobaric fragment [Tyr(-NH_3_)-Asp-Arg(-134-Adda)-Glu-Dhb(-CO)]. Extracted ion chromatograms for these MS/MS events demonstrates the utility of this transition in detecting [Mdha^7^] versus [Dhb^7^] isomers.

## 3. Discussion

MCs containing Dhb^7^ were observed from the cyanobacteria *Planktothirix* and *Nostoc* spp. [22,23,24,25]. The sample in this study was from an impoundment fed from Oser and Bischoff reservoirs in 2018 where the two dominant forms of cyanobacteria present were *Planktothrix agardhii* and *Pseudanabaena limnetica*, which are known producers of MCs [26]. Analysis of the sample determined the presence of [d-Asp^3^]-MC-RR and MC-LY as well as two untargeted MCs, [Dhb^7^]-MC-YR and [d-Asp^3^, Dhb^7^]-MC-LR. Furthermore, this is the first study to report the identification of [Dhb^7^]-MC-YR as well as the identification of Dhb MCs in the United States. Several *Planktothrix* sp. were reported in Europe and Japan with the main MCs produced being [d-Asp^3^] and [d-Asp^3^, Dhb^7^] MC congeners containing mainly arginine at position 4 [20,22,23,24,27]. Although *Planktothirx* and *Oscillatoria* spp. were identified in lakes throughout the United States in the US EPA National Lakes Assessments (2007 and 2012) [28,29], the [Dhb^7^] MC congener has not been reported in the US. These studies found that the occurrence of *Planktothirx* and *Oscillatoria* spp. was 38% [28], and they were the dominate cyanobacteria contributing to >1 ppb MC in three eco-regions (northern, temperate, and southern plains) [28,29], in which the current sample site was located. Other studies using cultures of *Planktothrix* sp. showed more diversity in MC congeners to include [d-Asp^3^] and [d-Asp^3^, Dhb^7^], containing the interchangeable fourth position MCs to contain amino acids other than arginine [20,30].

The online concentration LC–MS/MS analysis, quantitative selected reaction monitoring (SRM) and full scan, of the samples from the 2018 impoundment site detected four MCs initially determined to be [d-Asp^3^]-MC-RR, MC-YR, [d-Asp^3^]-MC-LR, and MC-LY. The three main MC congeners ([d-Asp^3^]-MC-RR, MC-YR, and [d-Asp^3^]-MC-LR) did not meet the more stringent retention time and/or quantifier/qualifier ion ratio (Table 1) criteria. Desmethyl MCs have several reported isomers, [DMAdda^5^], [d-Asp^3^], [Dha^7^], and [d-Asp^3^, Dhb^7^] [3,20,22,24]. LC–MS/MS analysis using water/acetonitrile (or water/methanol) with 0.1% formic acid solvent gradients showed that [DMAdda^5^], [d-Asp^3^], and [Dha^7^] MCs isomers are chromatographically discriminated. The DMAdda isomer elutes first and does not have the 135 *m/z* ion but rather a 121 *m/z* [3]. The [d-Asp^3^] and [Dha^7^] isomers have the 135 *m/z* fragment but have very unique products ions, since these units are far apart from each other in the structure. On the other hand, the [d-Asp^3^] and [d-Asp^3^, Dhb^7^] isomers chromatograph with similar/exact retention times and no reported unique product ions [20]. Both MC-YR and [d-Asp^3^]-MC-LR in the standard and impoundment samples had the designated quantifier (135 *m/z*) and qualifier (213 *m/z*) ions, which correspond to [Ph-CH_2_-CH(OCH_3_)] and [H + Glu-Mdha] or [H + Glu-Dhb], respectively. The qualifier/quantification transition ratios established by the method were 68% and 63% for MC-YR and [d-Asp^3^]-MC-LR, respectively (Table 1). The impoundment sample qualifier/quantification transition ratios were calculated to be 28% and 26% for MC-YR and [d-Asp^3^]-MC-LR, respectively, which was outside the ±25% window set within the method. The impoundment sample MC-YR and [d-Asp^3^]-MC-LR peaks visually showed lower peak height for the qualifier ion (213 *m/z*), suggesting that they have the same chemical formulas but different atom connectivity. The slight shift in chromatography and the monitored product ions suggest that (1) isomers of MC-YR, [d-Asp^3^]-MC-LR, and [d-Asp^3^]-MC-RR exist, and (2) for the 213 *m/z* product ion present for MC-YR and [d-Asp^3^]-MC-LR, the isomeric center is likely to be in position 7 as Dhb not Mdha. The change in the qualifier/quantifier (213 *m/z*/135 *m/z*) ratio observation could potentially be a mechanism to determine the presence of Dhb. Several of these trends were also observed by others who determined isomeric MCs [3,24,27]. Therefore, the three major MCs were suspect Mdha/Dhb isomers and were further investigated using an online concentration LC–MS/MS platform for chemical derivatization LC–MS/MS. Results were verified by precursor and product ion HRMS.

In order to advance freshwater HAB research, simple, robust, sensitive MS platforms need to be developed. Untargeted MC analytical platforms center around HRMS because of its ability to determine the chemical formulas of precursor and product ions with certainty [14,31]. Furthermore, chemical derivation with HRMS was used to determine if Mdha/Dha was present in the seventh position. The Dha chemical derivatization method was moved to a new platform, online concentration LC–MS/MS. The online concentrator dilutes 1 mL of sample with 0.1% formic acid water as it is injected onto the concentrator column while being cleaned with 0.1% formic acid in water during the 1-min injection period. This procedure was shown to remove unreacted reagents and buffers from the sample with little to no interferences observed [32]. Since the MCs in the sample were suspected to be Dhb isomers of the Mdha standards, the impoundment and standard samples were modified by chemical derivatization using 2-mercaptoethanol. This reagent reacts via nucleophilic addition with Mdha/Dha versus a minimal reaction with Dhb [14,19]. A 1.5-mL 50:50 mixture of derivatized and unreacted sample was analyzed by online concentration LC–MS full scan. Figure 2 shows the advantage of displaying them on the same chromatograph. The derivatization of the standard showed all of the MCs in the standard reacted except for [d-Asp^3^]-MC-RR (Figure 2a) and nodularin (data not shown), suggesting that neither compound contains Mdha. This was expected for nodularin because it contains an Mdhb group at position 5 [14]. When the impoundment sample was reacted with 2-mercaptoethanol, [d-Asp^3^]-MC-RR reacted, but MC-YR and [d-Asp^3^]-MC-LR did not (Figure 2b). The approximate concentration of the standards shown in Figure 2a was 50 ppt, and it was 75 ppt for the lowest MC in the impoundment sample ([d-Asp^3^]-MC-RR) (Figure 2b). These values highlight the advantage of using online concentration LC–MS with full scan due to its ability to measure low levels of MCs present in samples. Based on the derivatization of the impoundment sample, it contained two MCs without Mdha or Dha at the seventh position, one with the same nominal mass as MC-YR and the other with the nominal mass of [d-Asp^3^]-MC-LR. The derivatization of the standards suggests that standard [d-Asp^3^]-MC-RR is an MC without Mdha or Dha at the seventh position with the same nominal mass as [d-Asp^3^]-MC-RR.

HRMS techniques for accurate mass precursor and product ions were used to verify the results from online concentration LC–MS/MS showing that the impoundment sample had two Dhb^7^ MCs, [Dhb^7^]-MC-YR and [Asp^3^, Dhb^7^]-MC-LR. The calculated and experimental precursor accurate masses were compared and were considered matched if within 5 ppm, showing that the sample had isomers of the standards (Table 2). The search for unique fragments to help identify isomers of Mdha and Dhb gave two common fragments for each. Mdha MCs contained the unique fragments of [H + Mdha-Ala]+ (155.0815 *m/z*) and [Mdha-Ala-X-MAsp-Arg-C_11_H_14_O]^+^ (605.3280 + X *m/z*), and the Dhb isomers were found to contain two different fragments equal to 797.4192 *m/z* (Figure 4). Each MC fragmentation piece was identified within 7 ppm for each MC, which modeled the pieces of the molecule as a whole (Figure 3 and Supplementary Materials). Since the biosynthetic pathway is well described, the Dhb MC stereochemistries were presumed to be the same as known MCs. With the information provided by these complementary techniques, a new MC, [Dhb^7^]-MC-YR, was discovered, and the standard [d-Asp^3^]-MC-RR used for analysis was in fact [d-Asp^3^, Dhb^7^]-MC-RR. At present, *Microcystis* is not reported to produce Dhb-containing MCs; however, the manufacturers state that the MC standard assessed during the present study was produced using a *Microcystis* culture [3,14,19]. This raises the question of whether *Microcystis* sp. may be able to produce [d-Asp^3^, Dhb^7^]-MC-RR (and/or other Dhb-containing MCs), and highlights the importance of undertaking characterization work that discriminates between Mdha- and Dhb-containing MCs.

Two of the three major MCs in the sample had substitutions in the third and seventh positions, [d-Asp^3^] and [Dhb^7^]. [Dhb^7^] and [Mdhb^7^] are about two orders less reactive toward the addition of thiol-containing compounds than [Mdha^7^] and [Dha^7^] [19,20]. Protein phosphatase inhibition studies suggest that [d-Asp^3^, Dhb^7^] and [Dhb^7^] substitutions reduce protein phosphatase 2A inhibition up to one-fold [33]. The mechanism of MC cellular uptake occurs by active diffusion via organic anion transport protein 1 B1 (OATP1B1) and OATP1B3 [30]. A study designed to determine MC congener selectivity to OATP found that MC transport is favored by OATP1B3 and is more selective to the cyclo(-d-Ala^1^-l-Arg^2^-d-MAsp^3^–aromatic amino acid^4^–Adda^5^-d-Glu^6^-(*E*)-Dhb^7^ structure, which is similar to the most prevalent congener, [Dhb^7^]-MC-YR, reported in this paper [30]. Mouse LD_50_, through means of intraperitoneal injection, studies also suggested increased toxicity of Dhb-containing MCs. The mouse LD_50_ values for [d-Asp^3^, Dhb^7^]-MC-LR (70 µg/kg) [34] and [d-Asp^3^, Dhb^7^]-MC-RR (250 µg/kg) [34] were less than half of those for [d-Asp^3^]-MC-LR (160–300 µg/kg) [35] and MC-RR (500–800 µg/kg) [13], respectively. [d-Asp^3^, (*E*)-Dhb^7^]-MC-RR was also shown to be toxic to aquatic grazers with varying sensitivity [36,37]. An investigation with brine shrimp, *Artemia salina*, provided evidence that the varying sensitivity to [d-Asp^3^, Dhb^7^]-MC-RR may stem from glutathione *S*-transferase isoenzymes. The [d-Asp^3^, Dhb^7^] and [Dhb^7^] substitutions showed decreased 2-mercaptoethanol chemical reactivity and changed the spatial structure, providing mechanisms to alter in vivo uptake, distribution, metabolism, and excretion of MCs. With an increase in reported occurrences of [d-Asp^3^, Dhb^7^] and [Dhb^7^]-MC congeners [19,20,36,38], more toxicology research is needed to determine their environmental and human health impact.

## 4. Conclusions

Toxicokinetics and toxicodynamics were shown to be MC congener-dependent; thus, reliable platforms and workflows are needed to determine which MCs are most prevalent and their geographic distribution. This study developed a new platform, online concentration LC–MS/MS, to analyze the chemical derivatization of untargeted MCs. Online concentration LC–MS/MS in full scan mode detects derivatized MCs at the mid ppt level, minimizing sample preparation. This platform was used to investigate isomeric MCs. The chemical derivatization and HRMS results were used to verify that the standard [d-Asp^3^]-MC-RR, was probably [d-Asp^3^, Dhb^7^]-MC-RR and that the impoundment sample with *Planktothrix agardhii* present had two untargeted MCs, [d-Asp^3^, Dhb^7^]-MC-LR and tentative [Dhb^7^]-MC-YR, as the major components. Furthermore, this is first report of Dhb-containing MCs in United States. With three eco-regions in US reporting *Planktothrix* sp. blooms, this study supports the need (1) to integrate untargeted MC analyses in geographical studies, and (2) for certified reference standards to be readily available.

## 5. Materials and Methods

### 5.1. Chemicals and Reagents

Water, acetonitrile, methanol, acetic acid, and formic acid were all Optima LC–MS grade solvents purchased from Fisher Scientific (Tewksbury, MA, USA). Additionally, 2-mercaptoethanol was purchased from Sigma Aldrich (St. Louis, MO, USA). MCs MC-LR, RR, YR, WR, HtyR, HilR, [d-Asp^3^]-RR, [d-Asp^3^]-LR, LA, LF, LY, and LW, as well as nodularin, were purchased from Enzo Life Sciences, Inc. (Farmingdale, NY, USA). The surrogate C_2_D_5_ MC-LR was purchased from Cambridge Isotope Laboratories, Inc. (Tewksbury, MA, USA). The Microcystins-Adda ELISA kit was purchased from Abraxis, Inc. (Warminster, PA, USA). Standards were prepared in methanol and diluted in LC–MS grade water. Samples were stored on ice after collection and until arrival to the laboratory where they were then frozen at −4 °C. Samples then went through a series of three freeze–thaw cycles before analysis [31,39,40].

### 5.2. Sample Collection

On 1 May 2018, a grab sample was taken from an impoundment fed by Oser and Bischoff Reservoirs, Indiana, USA. The sample was collected in a 500-mL bottle with no preservatives. The bottle was immediately placed on ice and stored at 4 °C until shipping. Samples were shipped overnight on ice to Lumigen Instrument Center, Wayne State University, Detroit MI within 24 h of collection. The samples underwent three freeze–thaw cycles prior to analyses or concentration. On the day of analysis, the thawed sample was centrifuged at 10,000 rpm for 10 min at 4 °C, and the supernatant was collected and analyzed.

### 5.3. ELISA

Samples were tested using the Abraxis Adda-ELISA following procedures provided by the kit and as described in Birbeck et al. (2019) [32]. The ELISA kit was stored at 4 °C until used. The plate was read with a SpectraMax M2e microplate reader (Molecular Devices, LLC. San Jose, CA, USA) at 450 nm.

### 5.4. Online Concentration Liquid Chromatography Mass Spectrometry

Samples were analyzed by an online concentration method set up in house using a Thermo Scientific TSQ Altis™ triple quadrupole mass spectrometer (Thermo Scientific, Waltham, MA, USA) with an EQuan MAX Plus™ system. The system was set up as described in Birbeck et al. (2019) [32]. Briefly, 1 mL of sample was injected onto a loading column (Thermo Scientific Hypersil GOLD aQ 2.1 × 20 mm, 12 μm particle size) using an HTC PAL autosampler (CTC Analytics, Zwingen, Switzerland) equipped with a 5-mL sampling syringe. The flow rate was 1.5 mL/min using 0.1% formic acid in water. Analytes were separated on a Thermo Accucore aQ, 50 × 2.1 mm, 2.6 µm particle size column at 35 °C. The analytical separation gradient consisted of 0.1% formic acid in water (mobile phase A), and 0.1% formic acid in acetonitrile (mobile phase B), at a flow rate of 0.5 mL/min. The gradient was set as follows: isocratic from 0–0.7 min at 24% B, then ramped to 26% B from 0.7–1.7 min, and then increased to 50% B from 1.7–5.5 min. The column was washed with 98% B from 5.51–6.5 min at a flow rate 1.0 mL/min, then equilibrated at 24% B from 6.5–8.5 min.

Mass spectrometry analysis was performed using an electrospray ionization source (ESI). The MS source settings were as follows: spray voltage set to 3500 V in positive ion mode, ion transfer tube temperature set to 325 °C, vaporizer temperature at 420 °C, sheath gas pressure at 50 units, auxiliary gas pressure at 10 units, sweep gas at one unit, collision gas at 1.5 mTorr, Q1 resolution set to 0.7 Da, and Q3 resolution set to 1.2 Da. A list of the SRM transitions for each standard can be found in Table 3. Quantitation data results were accomplished using TraceFinder™ EFS 4.1. Retention time, and quantitative and qualitative ion qualifications were set in TraceFinder™ and monitored to ensure that the proper MC was detected. Furthermore, a second method for full scan Q3 analysis was used. It was identical to the quantitative run for the LC gradient and MS source settings. The scan range was 300–1200 (*m/z*), the scan rate was set to 1000 (Da/s), and the Q3 resolution was 1.2 full width at half maximum (FWHM).

### 5.5. High-Resolution Mass Spectrometry

The MC isolates were analyzed using nano-LC–HRMS with a Thermo Easy-nano-LC 1200 Orbitrap LTQ-XL (Thermo Scientific, Waltham, MA, USA) by gradient elution through a Thermo peptide map C18 (100 Å, 50 μm × 15 cm column, 2 µm particle size). Mobile phase A was 0.1% formic acid water and mobile phase B was 0.1% formic acid in 80% acetonitrile in water. At time 0, mobile phase B was at 5% and it was increased to 25% at t = 30 min, followed by an increase to 35% at t = 40 min, and an increase to 95% at t = 50 min, where it was held for 10 min. A high-resolution scan was run using the Orbitrap followed by data-dependent analysis of the [M + 2H]^2+^ exact mass for the expected MCs. Target *m/z* values were fragmented by HCD set to 15 and CID at 35 with an isolation width of 2 *m/z*. Internal mass locks to background polysiloxanes and phthalates were used to improve mass accuracy. In fragmentation spectra, mass axis correction was determined by shifting to the assumption that the 135.08 peak was the common Adda fragment of 135.0804. The correction factor was applied to all reported masses to improve mass accuracy.

### 5.6. Solid-Phase Extraction

Solid-phase extraction (SPE) was performed on the samples using a Waters 6cc Oasis HLB cartridge (Waters Corporation, Milford, MA, USA). The column was conditioned with 7 mL of LC–MS grade methanol, then equilibrated with 7 mL of LC–MS grade water. Next, 100 mL of sample was brought through the column at <1 mL/min. Then, the column was rinsed with 7 mL of 5% methanol. A second rinse of 7 mL of 100% methanol was completed, and this rinse was collected. The rinses were evaporated under N_2_ gas. Samples were stored at −4 °C until ready for analysis.

### 5.7. Fractionation of SPE Concentrated Samples by HPLC-PDA

The concentrated samples were further separated and fractionated using a Waters Alliance 2695 HPLC with a Waters 996 photodiode array detector (Waters Corporation, Milford, MA). The samples were separated using gradient analysis on a Waters Atlantis dC18 OBD prep column (10 × 100 mm, 5 µm particle size). The mobile phases used for the gradient were 20 mM ammonium acetate in water (mobile phase A) and 100% methanol (mobile phase B), run as follows: 60% B from 0–1 min, then ramped to 75% B from 1–10 min, followed by column cleaning at 90% B from 16–18 min, and equilibration at 60% B from 18–21 min. The flow rate was set to 3 mL/min, and the column oven temperature was 35 °C. The fractionated samples were collected every minute and were evaporated using N_2_ gas and stored at −4°C until ready for analysis.

### 5.8. Chemical Derivatization of MCs

To test unknown MC structures, known standards and samples containing MCs were chemically derived with 2-mercaptoethanol. This procedure was used by others to determine unknown MCs, but was modified here to determine structures of known isomers of MCs [14,20,41]. The reaction proceeded as follows: 1 mL of the sample or standard (in water) was added to a vial, and 100 µL of 0.2 M sodium carbonate was added to the vial and vortexed to bring the pH to 9. Next, 5 µL of 2-mercaptoethanol was added to the sample or standard solution and was vortexed and left to sit capped in a hood. The reaction was stopped after 2 h by adding 10 µL of 1% acetic acid to the vial to bring the pH to 6. Control samples and standards were also prepared as above, but the addition of 2-mercaptoethanol was excluded.

## Figures and Tables

**Table 1:** Comparison of standard and sample ion ratio percent and retention times.

Analyte	Standard Ion Ratio (%)	Sample Ion Ratio (%)	Standard Retention Time (min)	Sample Retention Time (min)
[d-Asp^3^]-MC-RR	12	14	2.56	2.50
MC-YR	68	28	3.34	3.40
[d-Asp^3^]-MC-LR	63	26	3.60	3.59
MC-LY	75	84	4.64	4.64

**Table 2:** High-resolution MS results of the three prominent MCs found in the impoundment sample and the corresponding standard.

**Standard Analyte**	**Calculated Mass (*m/z*)**	**Found Mass (*m/z*)**	**Charge**	**Δ ppm**
[d-Asp^3^]-MC-RR	512.7824	512.7816	2	−1.5
MC-YR	523.2713	523.2715	2	0.4
[d-Asp^3^]-MC-LR	491.2738	491.2738	2	0
				
**Sample Analyte**	**Calculated Mass (*m/z*)**	**Found Mass (*m/z*)**	**Charge**	**Δ ppm**
[d-Asp^3^]-MC-RR	512.7824	512.7825	2	0.2
MC-YR	523.2713	523.2717	2	0.8
[d-Asp^3^]-MC-LR	491.2738	491.2740	2	0.4

**Table 3:** Online concentration analyte quantifier and qualifier ions and retention times.

Analyte	Quantifier Ion (*m/z*)	Qualifier Ion (*m/z*)	Retention Time (min)
[d-Asp^3^]-MC-RR	135.07	498.91	2.56
MC-RR	135.07	212.97	2.65
Nodularin	135.00	389.16	3.04
MC-YR	135.00	213.03	3.34
MC-HtyR	135.05	1031.46	3.44
MC-LR	135.07	155.08	3.56
[d-Asp^3^]-MC-LR	135.01	213.07	3.60
MC-HilR	135.00	155.08	3.82
MC-WR	135.03	626.25	3.93
MC-LA	776.41	372.16	4.44
MC-LY	868.42	494.18	4.64
MC-LW	517.18	446.17	5.35
MC-LF	852.41	478.17	5.49
C_2_D_5_ MC-LR	135.09	163.08	4.64

## Supplementary Materials

The following are available online at https://www.mdpi.com/2072-6651/11/11/653/s1: Figure S1: HRMS of the three main MCs found in the impoundment sample and the corresponding isomeric standard.
