# Supplementary material for: Dhb Microcystins Discovered in USA Using an Online Concentration LC–MS/MS Platform
Source: Toxins (Basel). 2019 Nov 10;11(11):653. doi: 10.3390/toxins11110653 (PMC6891738; doi:10.3390/toxins11110653)
Supplement: Supplementary file 1 [file toxins-11-00653-s001.pdf]

Supplementary Materials: Dhb Microcystins Discovered in USA Using an Online Concentration LC-MS/MS Platform

Johnna A. Birbeck, Nicholas J. Peraino, Grace M. O'Neill, Julia Coady and Judy A. Westrick

A [D-Asp^3]-MC-LR (standard)

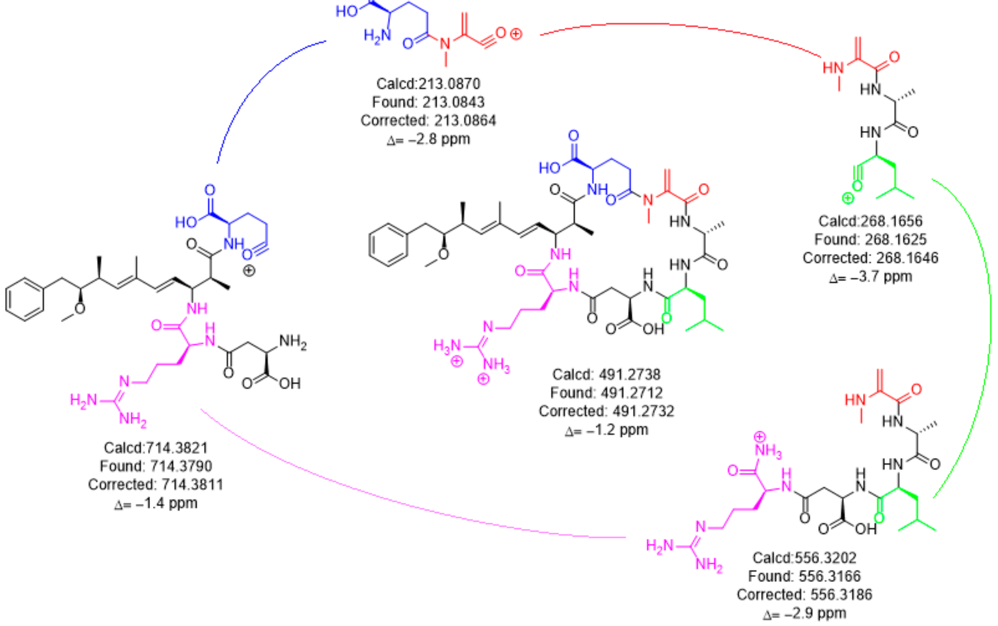

B [D-Asp^3, Dhb^7]-MC-LR (sample)

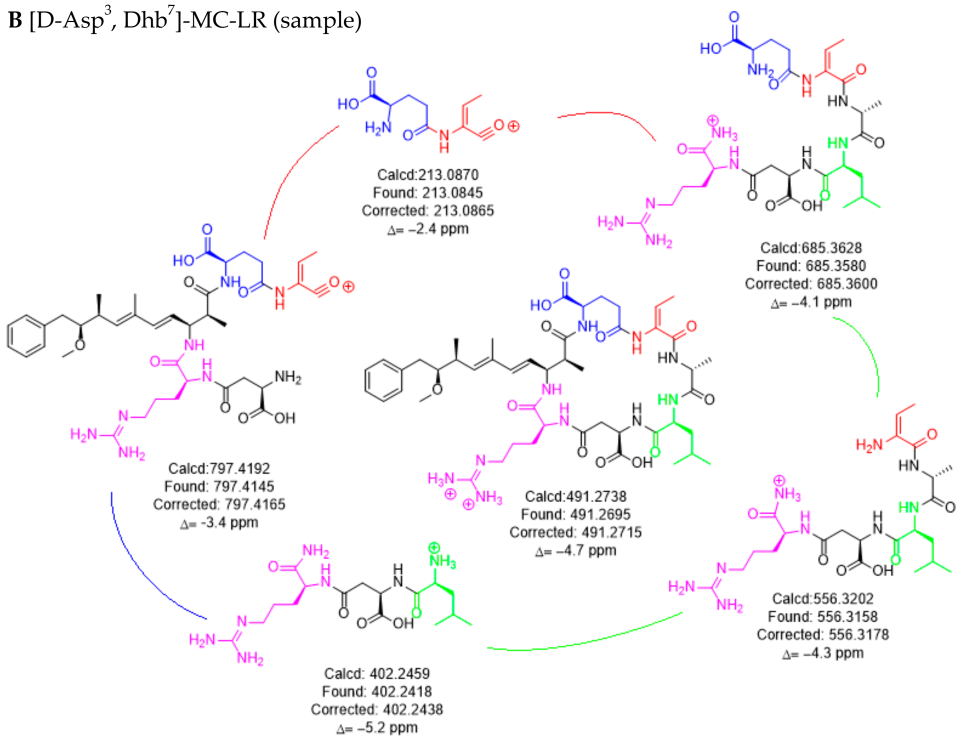

C MC-YR (standard)

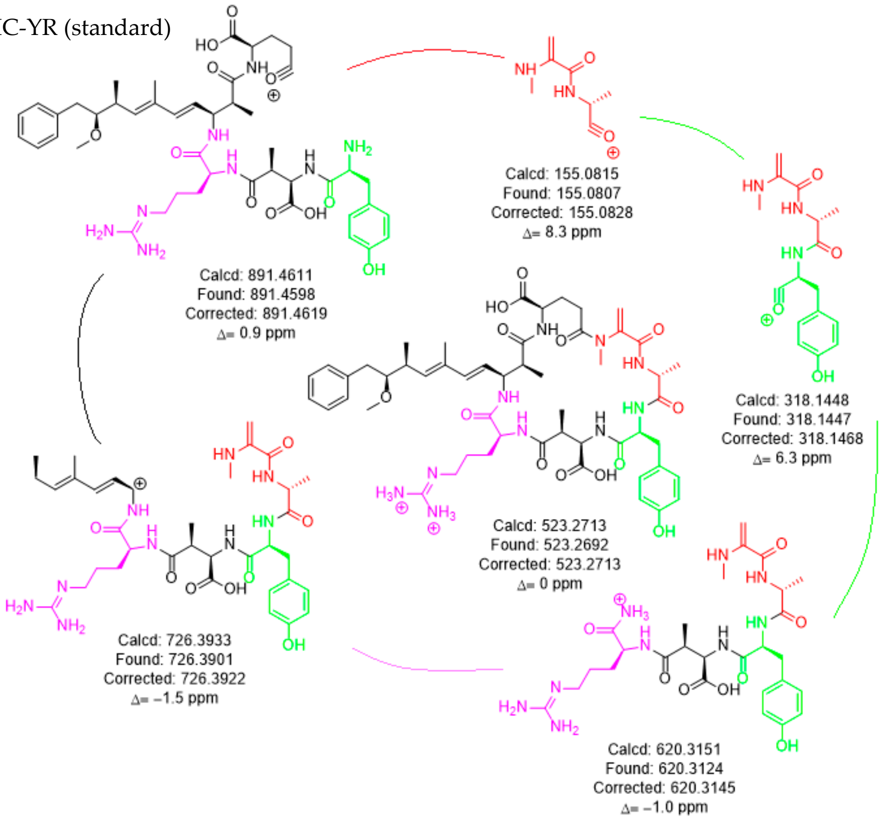

D [Dhb[7]]-MC-YR (sample) (figure 4)

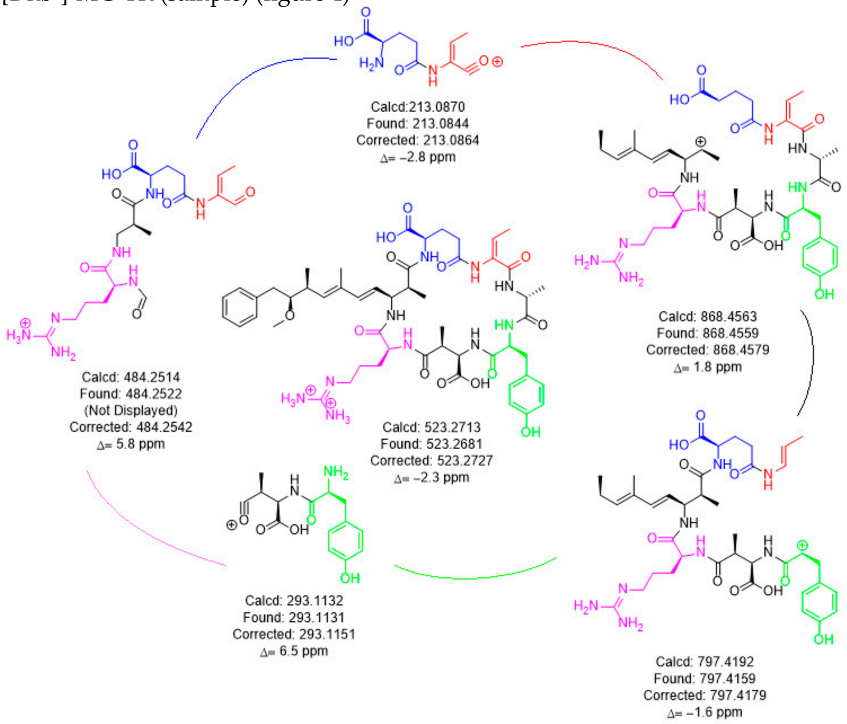

E [D-Asp^3]-MC-RR (sample)

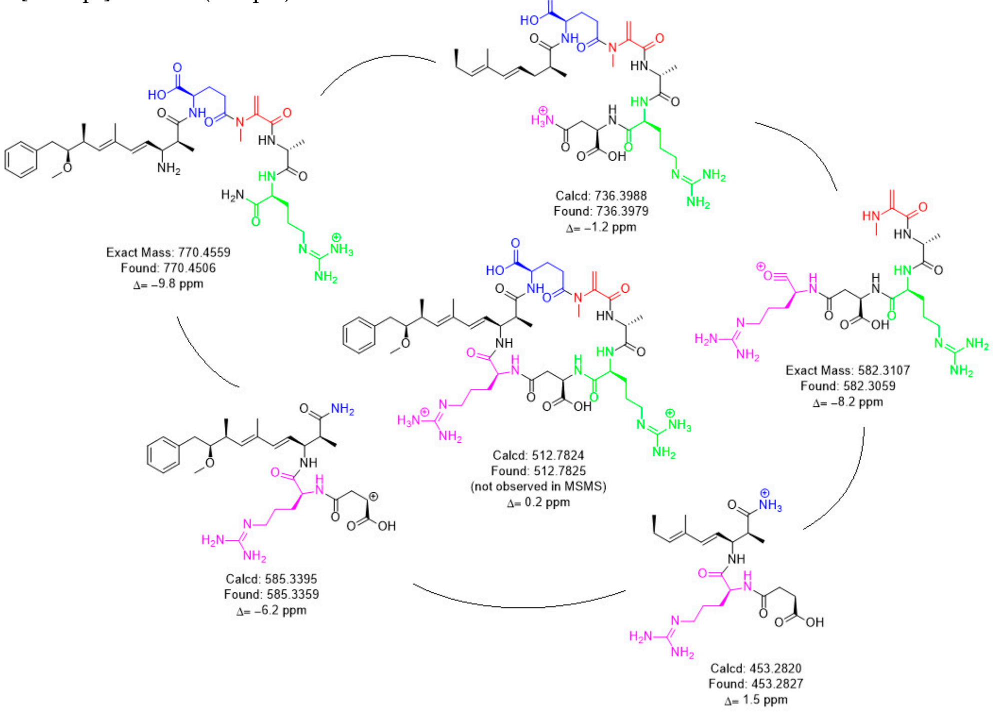

F [D-Asp^3, Dhb^7]-MC-RR (standard)

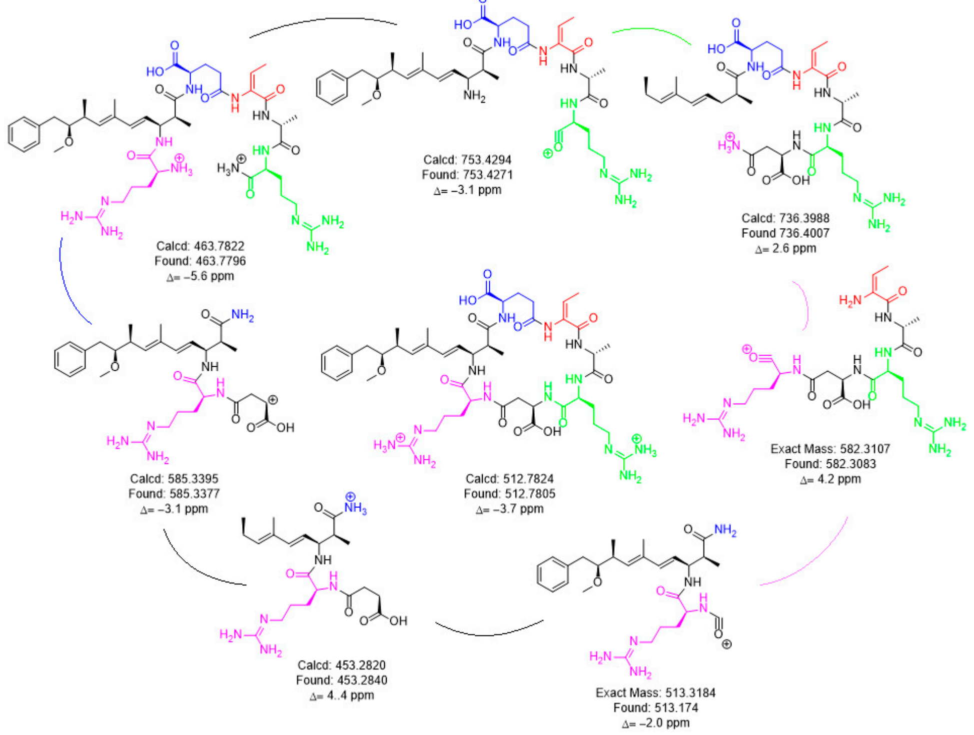

Figure S1. HRMS of the three main MCs found in the impoundment sample and the corresponding isomeric standard.
